# Targeted Metabolomic Serum Analysis of Patients with High and Low Risk of Endometrial Cancer Recurrence and Positive and Negative Lymph Node Status

**DOI:** 10.3390/metabo15070422

**Published:** 2025-06-20

**Authors:** Dagmara Pietkiewicz, Mikołaj Piotr Zaborowski, Szymon Plewa, Michał Potograbski, Cezary Miedziarek, Tomasz Kluz, Ewa Nowak-Markwitz, Jan Matysiak

**Affiliations:** 1 Doctoral School, Department of Inorganic and Analytical Chemistry, Poznan University of Medical Sciences, 3 Rokietnicka Street, 60-806 Poznan, Poland; dagmara.pietkiewicz@student.ump.edu.pl; 2 Gynecologic Oncology Department, Poznan University of Medical Sciences, 33 Polna Street, 60-535 Poznan, Poland; mzaborowski@ump.edu.pl (M.P.Z.); mpotograbski@ump.edu.pl (M.P.); cezary.miedziarek@gmail.com (C.M.); ewamarkwitz@ump.edu.pl (E.N.-M.); 3 Institute of Bioorganic Chemistry, Polish Academy of Sciences, Zygmunta Noskowskiego 12/14, 61-704 Poznan, Poland; 4 Department of Inorganic and Analytical Chemistry, Poznan University of Medical Sciences, 3 Rokietnicka Street, 60-806 Poznan, Poland; 5 Department of Gynaecology, Gynaecologic Oncology and Obstetrics, Institute of Medical Sciences, Medical College of Rzeszow University, Rejtana 16c Street, 35-959 Rzeszow, Poland; jtkluz@interia.pl

**Keywords:** metabolomics, endometrial cancer, biomarkers, lipids, amino acids

## Abstract

Background: Endometrial cancer is among the most prevalent gynecological malignancies, with increasing mortality primarily due to initially advanced disease with lymph node metastasis or tumor recurrence. Current risk stratification models show limited accuracy, highlighting the need for more accurate biomarkers. This study aimed to identify metabolic compounds that can serve as predictors of recurrence risk and lymph node status in endometrial cancer. Methods: Targeted metabolomic profiling of preoperative serum samples from 123 patients with endometrial cancer, stratified into high- or low-risk and lymph node-positive or -negative groups, was conducted using the AbsoluteIDQ p180 Kit and high-performance liquid chromatography–mass spectrometry. Results: Analysis revealed significant differences in metabolites related to lipid and amino acid metabolism between groups. High-risk and lymph node-positive patients presented significantly lower concentrations of phosphatidylcholines, lysophosphatidylcholines, medium-chain acylcarnitines, and specific amino acids such as alanine, histidine, and tryptophan compared to low-risk and lymph node-negative patients. Receiver operating characteristic curve analyses highlighted the diagnostic potential of these metabolites, particularly alanine and taurine, in distinguishing patient groups. Conclusions: The findings indicate complex metabolic reprogramming associated with aggressive endometrial cancer phenotypes, involving enhanced lipid utilization and amino acid metabolism alterations, potentially supporting tumor proliferation and metastatic progression. Thus, targeted metabolomic serum profiling might be a powerful tool for improving risk assessment, enabling more personalized therapeutic approaches and management strategies in endometrial cancer.

## 1. Introduction

Endometrial cancer (EC) stands as the most prevalent gynecological malignancy in women worldwide. EC is among the cancers with increasing mortality. The death rate has risen by 1.7% per year since the mid-2000s [1]. Although a significant number of ECs are diagnosed at an early stage and successfully treated without recurrence, many patients exhibit high-risk features that correlate with an increased probability of cancer recurrence and a poorer overall outcome [2]. The ESGO–ESMO–ESTRO risk stratification model might be a useful framework for guiding treatment decisions in EC. Its accuracy, especially when used preoperatively, appears to be only moderate and may be enhanced by integrating molecular and immunohistochemical data. Therefore, further refinement is needed [3,4] and identifying new biomarkers is needed to facilitate more precise risk assessments and maximize the efficacy of patient management strategies.

Lymph node metastasis (LNM) is recognized as one of the most important prognostic indicators in EC. LNM indicates a more advanced stage of the disease and is strongly linked to a higher risk of both local and distant recurrence. Many studies have shown that patients with LNM have significantly worse outcomes compared to those who do not have lymph node involvement [3,4,5]. Systemic lymph node dissection bears significant side effects, such as lymphedema. Therefore, assessing the risk of nodal metastasis without the need to resect all lymph nodes might be a beneficial approach for a patient with EC. Recent studies have focused on developing predictive models that integrate molecular data with clinical parameters to assess the risk of LNM preoperatively. It has been highlighted that integrating clinical, molecular, and imaging information is crucial for refining LNM prediction in EC [6,7].

It has been revealed that the prognosis of patients with EC is related especially to histopathological type, molecular profile, and pathological features. Dividing patients with EC into low- and high-risk groups has a significant influence on postoperative adjuvant therapy, including radiation, chemotherapy, and immunotherapy. Identifying patients with high-risk disease is unclear. Criteria for a group of patients with high-risk disease, who achieved an improvement in overall survival with chemoradiotherapy versus radiotherapy alone, were described in the PORTEC-3 study [8]. The high-risk group included FIGO stage IA grade 3 with lymphovascular space invasion; stage IB grade 3 disease; stage II disease; stage IIIA, IIIB, and IIIC disease; or stage IA–III with serous or clear cell histology. Despite advances in clinical risk stratification systems, their predictive accuracy for recurrence and lymph node metastasis remains suboptimal. This underlines the need for alternative approaches, such as metabolomic profiling, to improve risk assessment and guide personalized management strategies.

Metabolomics, an emergent and rapidly expanding discipline within systems biology, has gathered attention as a promising avenue for biomarker discovery in oncology [9,10]. By enabling the comprehensive analysis of small-molecule metabolites, the end products of cellular biochemical processes, metabolomic profiling provides an integrated snapshot of the interplay between genomic, transcriptomic, and proteomic alterations in cancer cells, which can significantly enhance opportunities for early diagnosis, prognosis, and the monitoring of therapeutic responses [11]. In the context of EC, there is an increasing acknowledgment of the metabolic reprogramming that occurs during tumor growth and recurrence, revealing notable changes in lipid metabolism, amino acid metabolism, and energy homeostasis, all of which may contribute to disease progression and patient outcomes [12,13].

Targeted metabolomics focuses on quantifying essential metabolites using high-performance analytical techniques, such as high-performance liquid chromatography–mass spectrometry (HPLC-MS), significantly enhancing biomarker detection’s sensitivity and reproducibility [14,15]. Recent advancements in metabolomic studies have successfully differentiated malignant endometrial tissue from benign conditions, highlighting its potential utility in clinical applications [16,17,18]. There remains a significant gap in the literature regarding the specific metabolic differences between patients with high-risk and low-risk EC, especially when evaluating their risks of recurrence. In the current study, a comprehensive targeted metabolomic analysis was conducted utilizing preoperative serum samples. Patients were postoperatively classified into high- and low-risk and lymph node-positive (LNP) and lymph node-negative (LNN) groups based on established clinicopathological criteria. This study aimed to unveil potential metabolic biomarkers relevant to recurrence risk by utilizing the AbsoluteIDQ p180 Kit (Biocrates Life Sciences AG, Innsbruck, Austria). By identifying disease-specific metabolic patterns, this study aims to enhance the development of prognostic tests and to refine personalized treatment strategies that could significantly improve care and outcomes for patients diagnosed with EC.

## 2. Materials and Methods

### 2.1. Study Groups and Sample Collection

Serum samples from 123 patients with malignant uterine cancer were collected at the Clinical Gynecology and Obstetrics Department of the Wojewódzki Szpital nr 1 im. Fryderyk Chopin in Rzeszów in 2016–2017. All patients provided signed informed consent approved by the Ethics Review Board of the Medical Chamber in Rzeszów (Consent No. 90/B/2016). Epidemiological and clinical characteristics characterizing patients are presented in Table 1.

Blood samples were obtained from individuals diagnosed with EC before surgery based on endometrial biopsy results. To minimize pre-analytical variability, all blood samples were centrifuged within 1 h of collection, aliquoted, and stored at −80 °C until analysis. Inclusion in the study required a confirmed diagnosis of EC. Patients with EC were categorized into low- and high-risk groups and LNN and LNP groups. The high-risk group included patients with FIGO stage IA grade 3 with lymphovascular space invasion; stage IB grade 3; stage II; stage IIIA, IIIB, or IIIC; or stage IA–III with serous or clear cell histology. All other patients were classified as low-risk. Patients underwent laparoscopic or abdominal hysterectomy with sentinel lymph node dissection or systemic lymphadenectomy. Patients with confirmed LNM were classified in the LNP group, and all other patients were classified as LNN.

### 2.2. Sample Preparation

The AbsoluteIDQ p180 Kit (Biocrates Life Sciences AG, Innsbruck, Austria) was used for serum metabolomic profiling. The kit allows for the simultaneous determination of 145 lipid metabolites: 40 acylcarnitines, 15 sphingomyelins, 90 glycerophospholipids (14 lysophosphatidylcholines (lysoPCs) and 76 phosphatidylcholines (PCs)), 22 amino acids, 23 biogenic amines, and hexoses (including glucose). The samples were prepared according to the manufacturer’s protocol. All assays were carried out on a 96-well plate. Lyophilized internal standards (ISTDs) were dissolved in 1200 µL of water, shaken for 15 min at 1200 rpm, and vortexed several times. Calibration standards were reconstituted with 100 µL of water each, shaken for 15 min at 1200 rpm, and vortexed several times. To each quality control (QC) sample, 100 µL of water was added. The QCs were shaken for 15 min at 1200 rpm and vortexed several times. Next, QCs were centrifuged at 4 °C for 5 min at 2750× *g* before loading onto the kit plate. Serum samples were vortexed after thawing and centrifuged at 4 °C for 5 min at 2750× *g*. Initially, 10 µL of ISTD solution was added to each well of the 96-well kit plate, except for the designated blank well. Subsequently, the plate was subjected to a drying step under a controlled nitrogen flow for 30 min to remove residual solvents. To facilitate derivatization, 50 µL of a freshly prepared 5% phenyl isothiocyanate solution was pipetted into each well, and then incubated at room temperature for 25 min. After derivatization, an additional drying step was conducted for 60 min under nitrogen to ensure complete evaporation of excess reagents. Metabolites were extracted by adding 300 µL of an extraction solvent containing 5 mM ammonium acetate in methanol to each well, with subsequent shaking for 30 min. The resulting extracts were then eluted under nitrogen pressure and collected for further processing. To optimize detection sensitivity, the extracts were split into two separate plates for liquid chromatography–mass spectrometry (LC-MS) and flow-injection analysis–mass spectrometry (FIA-MS), followed by appropriate dilution steps. Then, 150 µL of water was added to each well of the LC-MS plate, and 400 µL of FIA solvent was added to each well of the FIA plate. Subsequently, 5 min of shaking at 600 rpm was applied to both plates. Finally, the prepared plates were placed directly into the autosampler for immediate analysis.

### 2.3. High-Performance Liquid Chromatography–Mass Spectrometry Analysis

All samples were randomized before the analysis. A triple-quadrupole tandem mass spectrometer 4000 QTRAP (Sciex, Framingham, MA, USA) and a high-performance liquid chromatograph 1260 Infinity (Agilent Technologies, Santa Clara, CA, USA) were utilized. The samples were analyzed using LC and FIA in multiple reaction monitoring mode. The LC system was operated with a column oven temperature set at 50 °C to maintain consistent chromatographic conditions. The gradient elution method employed a mobile phase consisting of solvent A (water with 0.2% formic acid) and solvent B (acetonitrile with 0.2% formic acid), with a flow rate of 0.8 mL/min adjusted throughout the analytical run. The injection volume was 10 µL for high-performance liquid chromatography (HPLC). For FIA, a fixed injection volume of 20 µL was used to ensure consistent ionization conditions across samples. FIA was performed under gradient conditions using methanol with a mobile phase additive from the kit. The mass spectrometric detection utilized multiple reaction monitoring (MRM) in positive and negative ionization modes, with scheduled MRM detection windows set at 30 s to enhance sensitivity and minimize background noise. Data acquisition was performed using Analyst software, with retention time adjustments performed before final quantification to ensure peak accuracy.

The methodology was verified using QC samples at 3 concentration levels provided in the kit and injected throughout the sequence. All the measured analytes passed quality control. The intra-assay coefficient of variation was calculated from the QC sample repetitions and was 3.7%.

### 2.4. Data Analysis

WebIDQ (Biocrates Life Sciences, AG, Innsbruck, Austria) was used for automated quantification, integration, and calibration. Quality control assessments ensured the validity of calibration standards, internal controls, and sample measurements. The concentrations of all analyzed metabolites were automatically calculated and are displayed in micromolar (µM). For data cleaning, the cleaning threshold was set to 70%, and as a result, the number of metabolites was reduced to 138. Missing values were imputed using the K-nearest neighbors (KNN) method. The list of all 138 metabolites, along with QC metrics and limits of detection, is presented in the Supplementary Material (Table S1). Normalization of metabolomic data was performed to ensure accuracy, comparability, and reproducibility across two analytical runs. Internal standards and QC samples were used to correct technical variations introduced during sample preparation and mass spectrometric analysis. The normalization process included the analysis of QC level 2 (QC2) samples in replicates of five, evenly distributed across each analytical plate. To control for both intra-plate and inter-plate variability, normalization was performed relative to the target values of QC2 samples. Additionally, a seven-point calibration curve was applied for LC data analysis, ensuring precise quantification of metabolites. For FIA, reference samples were employed to maintain consistency in metabolite measurements. Data normalization was conducted within WebIDQ software (v. DB134-792), following predefined algorithms that adjusted for batch effects and instrumental fluctuations. Statistical analysis was carried out using WebIDQ cloud-based software (Biocrates Life Sciences, AG, Innsbruck, Austria) and Metaboanalyst 6.0 [18]. Univariate analyses (ANOVA) were used to identify significant metabolites, and partial least squares discriminant analysis (PLS-DA) was employed for group separation. Receiver operating characteristic (ROC) curves were generated to evaluate the discriminatory performance of selected metabolites.

## 3. Results

A total of 123 samples of high- and low-risk EC were analyzed. The analysis of the obtained data showed significant differences between the analyzed groups. After Bonferroni correction, the univariate analysis of variance (ANOVA) test of all serum samples (*n* = 123) showed that the levels of 20 metabolites differed significantly between the compared groups of patients with a high and low risk of EC recurrence. Notably, alterations were observed in several lipid species, including phosphatidylcholines, lysophosphatidylcholines, acylcarnitines, and amino acids. In general, the high-risk group exhibited lower concentrations of these metabolites compared to the low-risk group. These findings indicate a broad metabolic distinction between the two groups, with high-risk patients showing a downregulation of key metabolites. The list of differentiating compounds is presented in Table 2.

**Figure 1:**
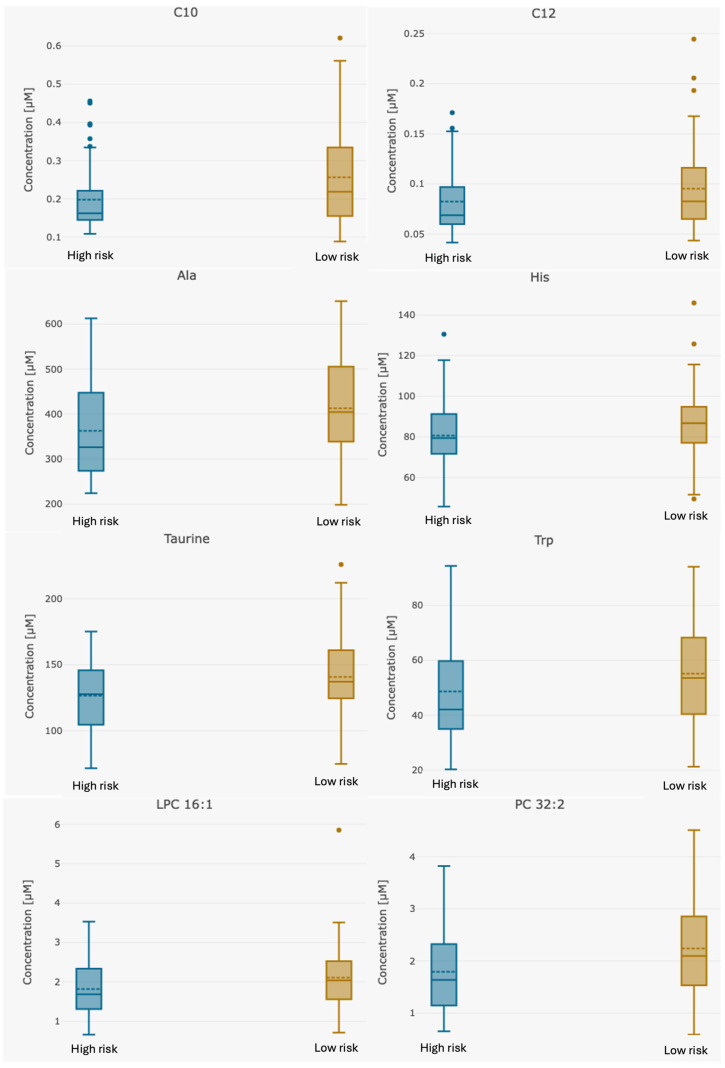

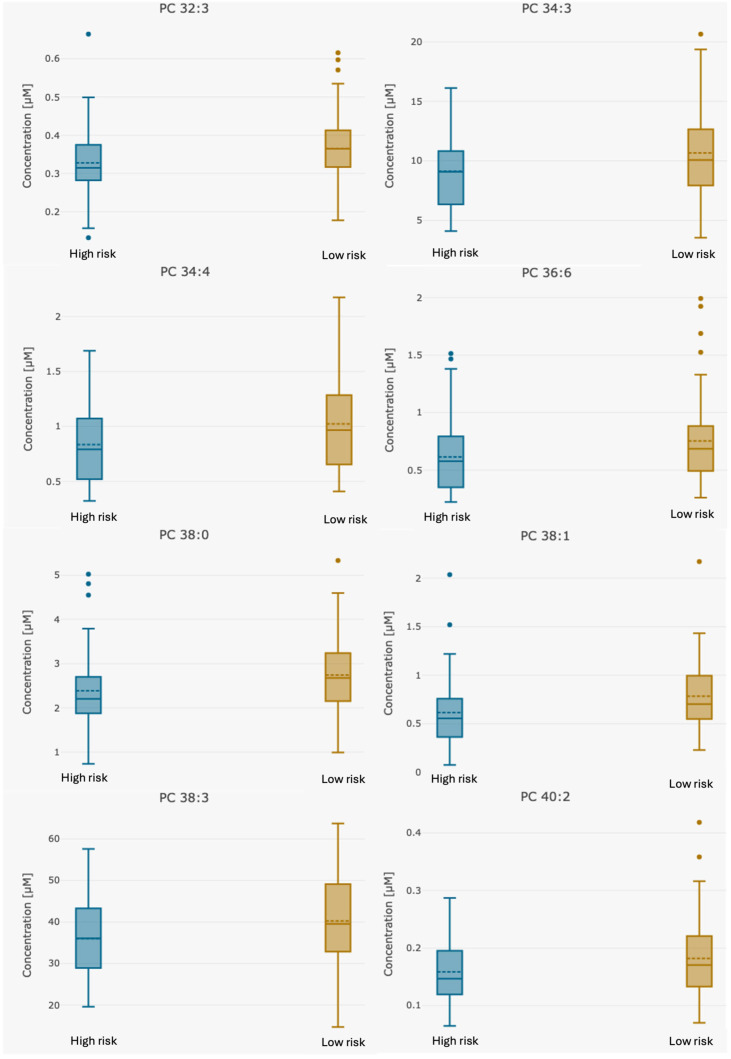

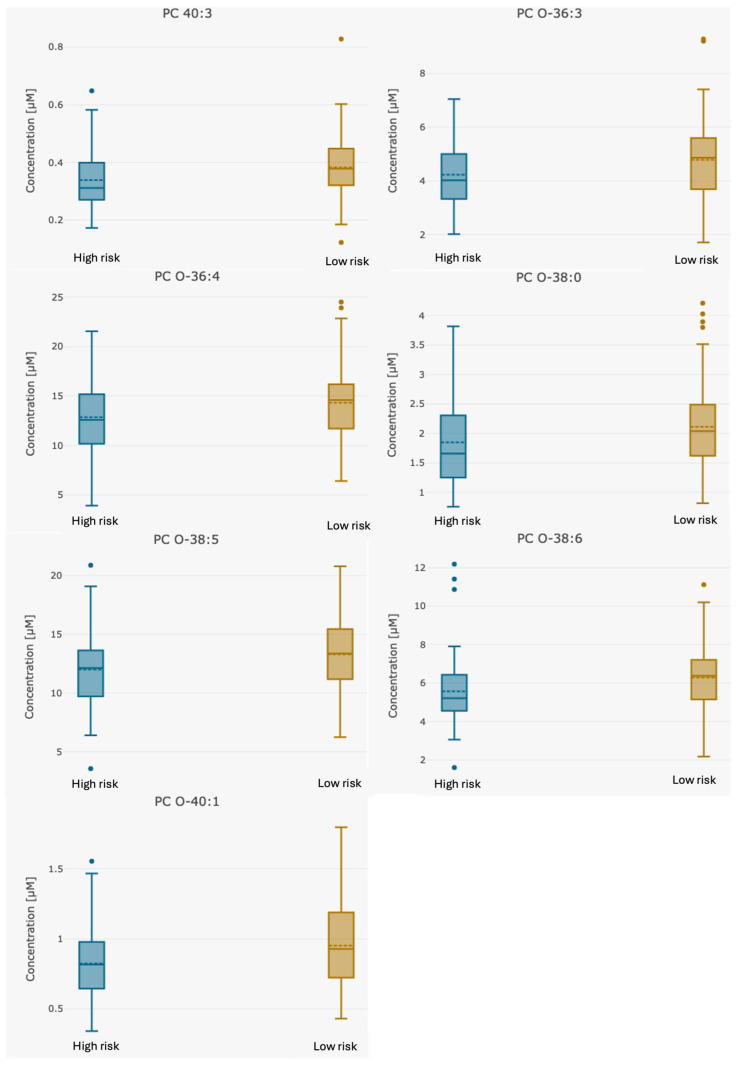
Box plots for differentiating compounds between high-risk (*n* = 55) and low-risk (*n* = 68) recurrence in patients with endometrial cancer. Abbreviations: Ala—alanine, C10—decanoylcarnitine; C12—Lauroyl-L-carnitine; LPC—lysophosphatidylcholine; His—histidine; PC—phosphatidylcholine; Trp—tryptophan.

**Figure 2:**
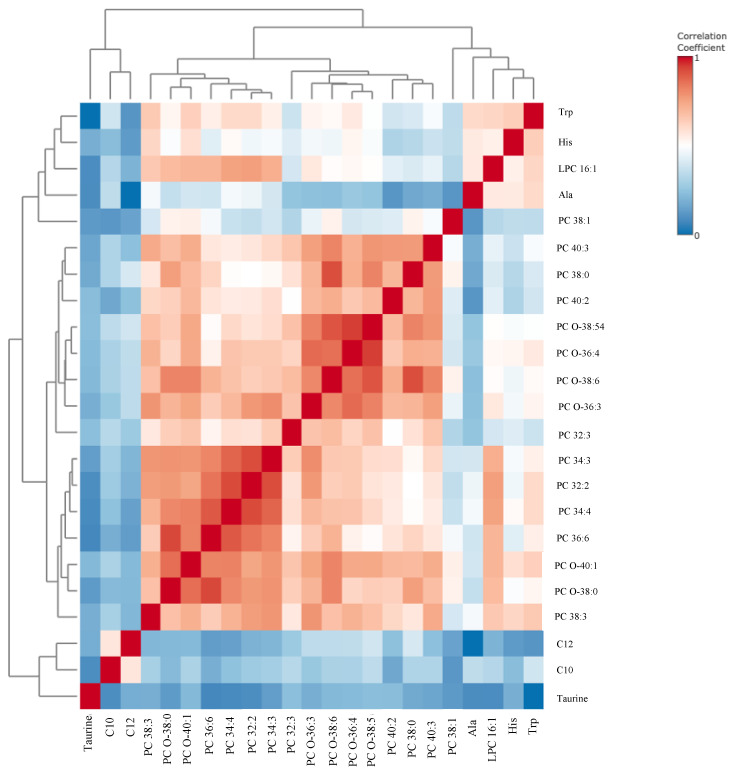
Hierarchical clustering correlation heatmap for differentiating compounds between patients with high risk (*n* = 55) and low risk (*n* = 68) of endometrial cancer recurrence. Abbreviations: Ala—alanine, C10—decanoylcarnitine; C12—Lauroyl-L-carnitine; LPC—lysophosphatidylcholine; His—histidine; PC—phosphatidylcholine; Trp—tryptophan.

Box-and-whisker plots (Figure 1) and a correlation heatmap (Figure 2) were created to visually represent data distribution. The median metabolite levels for high-risk patients are consistently lower than those of low-risk patients. The interquartile ranges (IQRs) in the high-risk patients are of similar width or slightly narrower, suggesting that metabolite levels are uniformly suppressed across these patients.

Figure 2 displays a hierarchical clustering correlation heatmap of 20 metabolites that distinguish between high-risk and low-risk patients for EC recurrence. The heatmap reveals strong positive intercorrelations within the phosphatidylcholine cluster. Nearly all PCs tend to increase or decrease together, indicating that they are co-regulated. Additionally, moderate positive correlations are observed between certain amino acids and specific phosphatidylcholine species. This suggests that common metabolic pathways or cancer-driven metabolic changes may link these amino acids and phospholipids. The medium-chained acylcarnitines showed modest correlations with specific phospholipids and amino acids, implying a partial coupling of fatty acid β-oxidation intermediates with amino acid and lipid metabolism.

**Figure 3:**
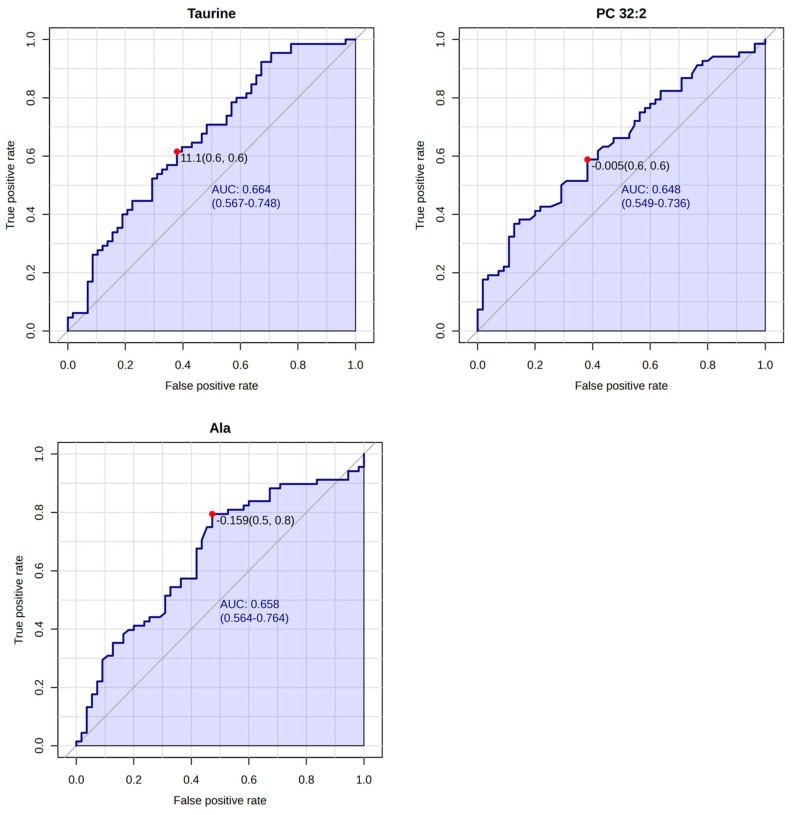
ROC curves with the highest AUC (area under the curve) values for the differentiating metabolites between high-risk (*n* = 55) and low-risk (*n* = 68) patients. Abbreviations: Ala—alanine; AUC—area under curve; PC—phosphatidylcholine; ROC—receiver operating characteristic.

The diagnostic utility of the selected metabolites was further evaluated by ROC curve analysis. Overall, the metabolites showed moderate discrimination between high- and low-risk patients. The highest area under the ROC curve values were found for taurine, PC 32:2, and alanine (Figure 3). Taurine showed the greatest separation of high- and low-risk patients. Alanine and PC 32:2 had similar performance, reflecting the overlapping contributions of amino acids and lipid alterations to recurrence classification.

The univariate ANOVA test performed for the LNN and LNP groups showed significant differences between the compared groups of patients. The levels of 15 metabolites were significantly different (7 out of the 15 differentiating metabolites were amino acids, and the remaining 8 were PCs). These differences highlighted changes in both amino acids and lipid metabolism in patients with lymph node involvement. All 15 metabolites showed decreased concentrations in the LNP group compared to the LNN group. The list of differentiating compounds is presented in Table 3.

**Figure 4:**
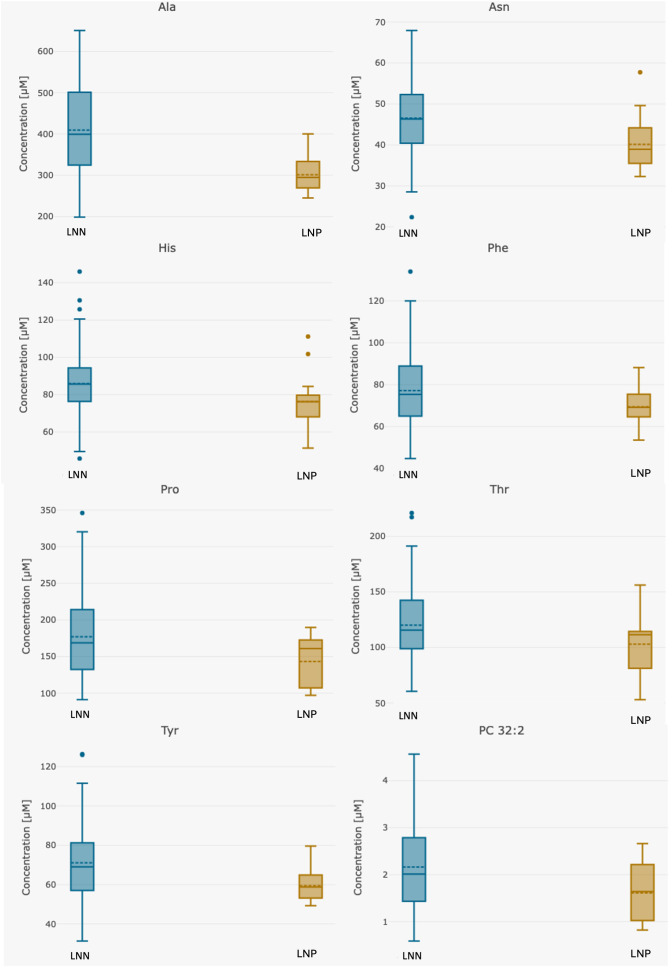

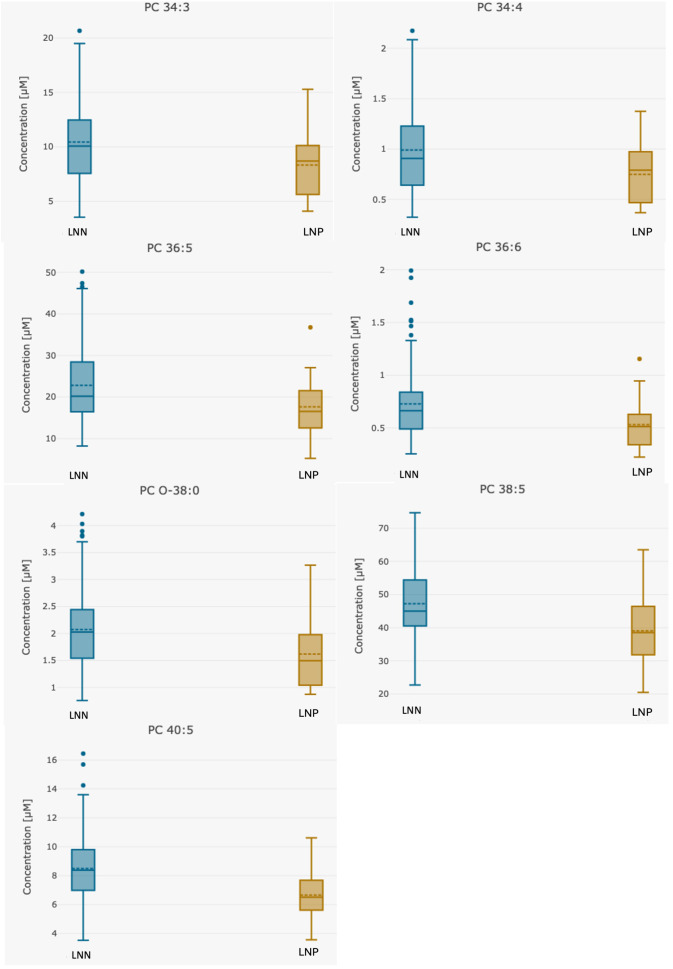
Box plots for differentiating compounds between LNN (*n* = 107) and LNP (*n* = 16) patients. Abbreviations: Ala—alanine, Asn—asparagine; His—histidine; LNN—lymph node-negative; LNP—lymph node-positive; PC—phosphatidylcholine; Phe—Phenylalanine; Pro—Proline; Thr—Threonine; Tyr—Tyrosine.

The distribution of these metabolites was visualized in box plots (Figure 4). The narrower IQRs observed in the lymph node-positive group may reflect a more uniform metabolic phenotype. However, the small sample size could also contribute to reduced variability.

**Figure 5:**
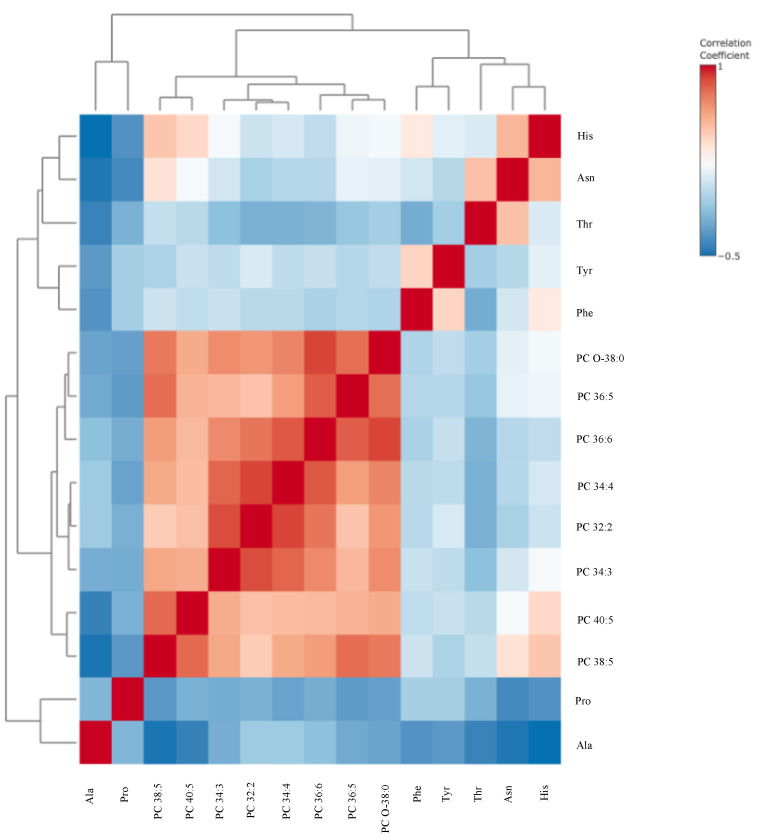
Hierarchical clustering correlation heatmap for differentiating compounds in LNP (*n* = 16) and LNN (*n* = 107) patients with endometrial cancer. Abbreviations: Ala—alanine; Asn—asparagine; His—histidine; LNN—lymph node-negative; LNP—lymph node-positive; PC—phosphatidylcholine; Pro—Proline; Thr—Threonine; Tyr—Tyrosine.

For a visual representation of correlations between differentiating metabolites, a hierarchical clustering heatmap was used (Figure 5). Two clusters are evident: one comprised amino acids and another comprised PCs. Metabolites within each cluster show strong intracluster correlations but with weaker intercluster correlations. Cross-talk between the amino acid cluster and PC cluster appears to be limited.

**Figure 6:**
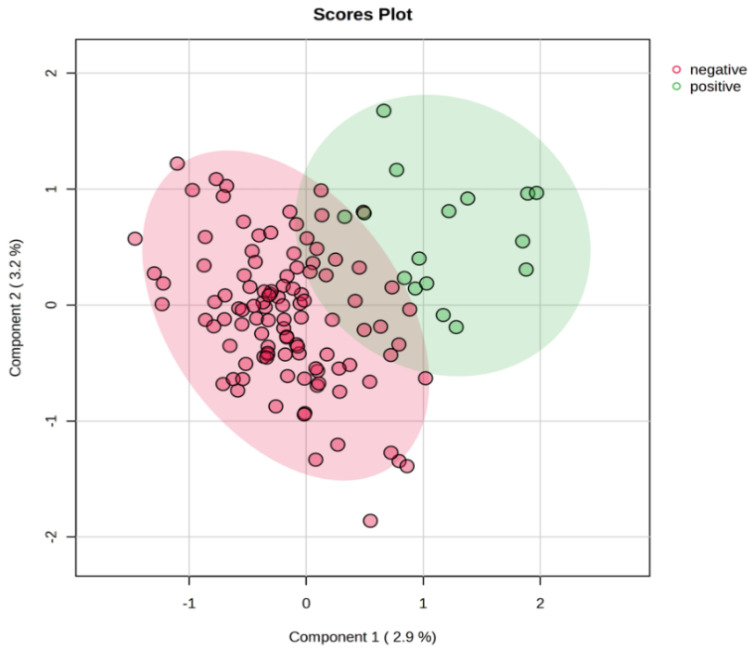
PLS-DA plot showing separation between LNP (*n* = 16) (positive) and LNN (*n* = 107) (negative) groups. LNN—lymph node-negative; LNP—lymph node-positive; PLS-DA—partial least squares discriminant analysis.

For the visualization of the separation between the LNN and LNP groups, PLS-DA was performed (Figure 6). The compared groups were separated with limited overlap, which indicates that the metabolic profiles of these two groups are distinct. Despite the relatively low percentage of variance explained by the two components presented (2.9% and 3.2%), the separation between groups suggests potential biological differences. However, due to the low variance explained, these findings should be interpreted with caution.

**Figure 7:**
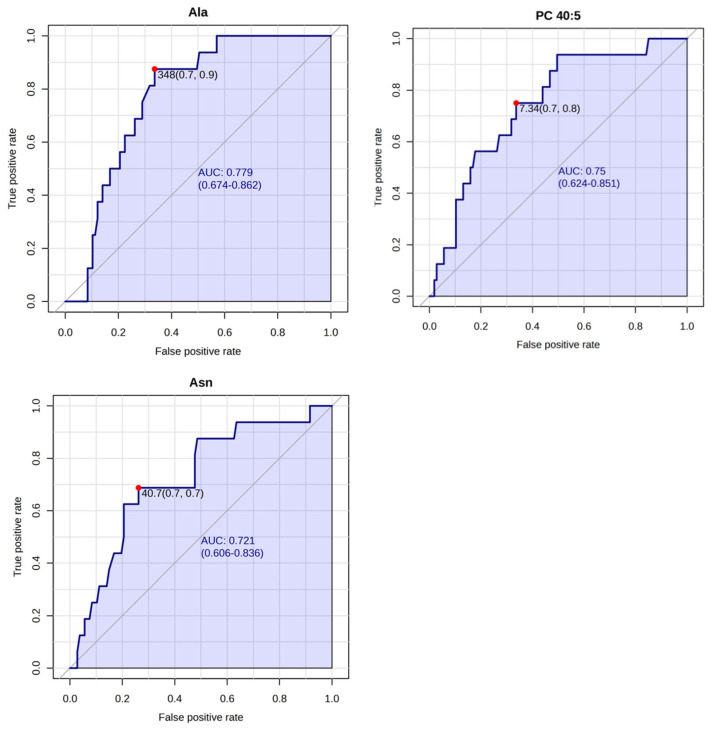
ROC curves with the highest AUC (area under the curve) values for differentiating metabolites between LNP (*n* = 16) and LNN (*n* = 107) patients. Abbreviations: Ala—alanine; Asn—asparagine; AUC—area under curve; LNN—lymph node-negative; LNP—lymph node-positive; PC—phosphatidylcholine; ROC—receiver operating characteristic.

ROC curve analysis for the LNN and LNP groups demonstrated better diagnostic performance of metabolites than seen for recurrence risk (Figure 7). Alanine, PC 40:5, and asparagine achieved the highest AUC for distinguishing between LNN and LNP patients.

## 4. Discussion

In this study, targeted metabolomics was applied to serum samples from patients with EC, stratified by recurrence risk and lymph node status, to characterize the metabolic alterations associated with aggressive disease. Our data revealed that patients classified as high-risk and those with lymph node metastasis exhibit significantly lower serum concentrations of key lipid species—PCs and LPCs—as well as reduced levels of certain amino acids such as alanine, histidine, and tryptophan. These metabolic changes likely reflect an increased tumor uptake of these metabolites or an upregulation of metabolic pathways that support rapid tumor proliferation and metastatic dissemination. These metabolic alterations align with previous studies that have reported lipid metabolism reprogramming as a hallmark of aggressive cancer phenotypes [19,20]. The reduction in PCs and LPCs observed in high-risk patients may indicate that tumor cells are actively channeling these lipids into membrane synthesis and energy production. This finding aligns with the broader concept of metabolic reprogramming in cancer, where lipid metabolism is altered to meet the demands of aggressive growth and metastatic potential [19,20]. In parallel, decreased levels of amino acids might reflect enhanced consumption by tumor cells to fuel energy production and anabolic processes, as described in ovarian cancers and ECs [21,22]. It should also be noted that hormonal factors, particularly estrogen and progesterone levels, can significantly influence lipid and amino acid metabolism [23]. Hormone-driven metabolic reprogramming may contribute to the differences observed between patient groups and should be considered in future studies.

The biological significance of depleting specific amino acids in aggressive EC is multifaceted. Tryptophan, for instance, is well known to be catabolized by the enzyme indoleamine-2,3-dioxygenase (IDO) in the tumor microenvironment, producing kynurenine and other metabolites that suppress immune responses. Consistent with our results, several studies that identified tryptophan as a discriminating metabolite in cancer have reported decreased tryptophan levels in patients [24,25]. Imbalances in tryptophan metabolism often correlate with poor prognosis by fostering tumor growth and immune evasion by creating an immunosuppressive tumor microenvironment [26]. In EC, high IDO expression and systemic tryptophan reduction have been associated with worse outcomes [27]. High IDO expression correlates with significantly fewer tumor-infiltrating T lymphocytes and NK cells. High-IDO-expressing tumors were associated with deep myometrial invasion, nodal metastasis, and worse progression-free survival [28]. Likewise, histidine was found at lower concentrations in the high-risk group. This decreased histidine concentration may be caused by tumors converting histidine into histamine, a bioactive amine that can stimulate tumor cell motility, angiogenesis, and invasion [24,27]. Several studies have noted that patients with EC often exhibit decreased histidine levels, hypothesizing that the tumor’s demand for histamine drives this decrease [24,29]. Alanine is a key link between glycolysis and the tricarboxylic acid (TCA) cycle via the alanine transaminase reaction. It can support cell growth during glucose withdrawal by supplying cells with a source of pyruvate to feed the TCA cycle and gluconeogenesis [30]. Many different tumors may present an increased uptake of alanine to support energetic and biosynthetic needs, which results in a decreased alanine concentration in blood [31]. In fact, some studies have found elevated alanine within tumor tissues, which supports the idea of increased alanine uptake and metabolism in cancers [32,33]. The analogous observations seem to be confirmed by Skorupa et al., who demonstrated increased concentrations of alanine and taurine in EC tissues compared to healthy tissues [34]. Convergent results obtained with two completely independent methodologies allow us to assume that the correlations found may be useful in differentiating patients’ conditions.

A key pathway in PC metabolism is the Kennedy pathway (CDP-choline pathway), which generates phosphatidylcholine de novo from choline. In EC, there is evidence that this pathway is upregulated within tumor cells. Specifically, aggressive endometrial tumors commonly overexpress choline kinase alpha (CHKA), the enzyme that phosphorylates choline in the first step of the Kennedy pathway [35]. CHKA overexpression leads to an accumulation of phosphocholine and drives enhanced PC biosynthesis. Indeed, altered choline phospholipid metabolism is a known feature of EC, with increased phosphocholine levels being the most significant lipid-related change in malignant endometrial tissue [35]. This is caused by both the overactive choline kinase and increasing PC production, and a “hyperactivated deacylation pathway,” wherein PC is more rapidly broken down [35]. This impacts circulating PC levels by decreasing them, as observed in patients with aggressive EC. Consistent with our findings, other studies highlight specific PCs as potential biomarkers in EC. For example, a study by Alberti-Valls et al. noted that the phosphatidylcholines (PCs) 40:1, 42:0, and 44:5 were significantly lower in patients with EC than in controls [36].

LPC levels are often dysregulated in patients with cancer. In patients with EC, a consistent finding is decreased LPC levels in patients’ blood [24]. In EC research, it was noted that a decrease in plasma LPC and PC concentrations may be due to increased LPC production in the tumor [24]. Studies show that this might be caused by the overexpression of autotaxin (ATX), especially in aggressive cancers, which leads to decreasing levels of LPCs in plasma and increasing lysophosphatidic acid (LPA) in the tumor microenvironment [37,38]. The same metabolomic pattern—low circulating LPCs and PCs—has been reported in patients with ovarian and cervical cancers, attributed to the tumor’s conversion of these lipids to LPA [24]. ATX is frequently upregulated in metastatic or high-grade tumors, and high ATX expression correlates with greater invasiveness and microvessel density [38]. In aggressive EC, the autotaxin–LPA axis contributes to tumor progression by fueling a pro-metastatic signaling loop: as LPA levels increase at the expense of LPC, LPA receptors on cancer cells drive processes like epithelial–mesenchymal transition, migration, and survival in the hostile circulation.

In the serum of patients with high-risk EC, medium-chain acylcarnitines (such as decanoylcarnitine C10 and dodecanoylcarnitine C12) were found at lower levels than in low-risk patients. This trend indicates alterations in fatty acid oxidation (FAO) activity in aggressive tumors or in the patients’ metabolism. Carnitine plays an important role in the transport of long-chain fatty acids into the mitochondrial matrix, and the ratio of short-chain acylcarnitines to free carnitine is a measure of overall ß-oxidation activity [39]. High FAO activity leads to lower acylcarnitine levels, as substrates are efficiently consumed by mitochondria. Recent studies indicate that aggressive forms of EC exhibit alteration in fatty acid metabolism, including increased FAO [40,41]. In a study by Arioza et al., it was noted that levels of L-carnitines tend to be lower with the progression of the disease [42]. These findings indicate that increased FAO and reduced acylcarnitine levels may contribute to the aggressiveness of certain ECs.

One of the limitations of our study is the relatively small size of the LNP group (*n* = 16), which may affect the statistical power of the results. Future studies with larger and independent cohorts are necessary to confirm and validate these findings. Additionally, due to the limited sample size and lack of an external validation cohort, we were unable to perform cross-validation or independent validation of the ROC and PLS-DA models. Also, potential confounding variables such as dietary habits, comorbidities like diabetes and obesity, and concomitant medication use may also impact serum metabolite profiles and represent limitations of the current study.

## 5. Conclusions

This study provides a novel serum metabolomic signature that differentiates patients with high and low recurrence risk as well as positive and negative lymph node status in EC. Our research indicates that patients with high-risk or LNP EC exhibit a distinct metabolic profile, characterized by reduced levels of certain lipids and amino acids. These alterations are likely due to increased tumor uptake and reprogramming of systemic metabolism in favor of cancer progression. The observed correlations among lipids and associations with amino acids not only provide insight into the underlying biochemical pathways but also suggest that an integrated metabolite panel will better define risk assessment and personalized treatment. These findings highlight the potential utility of metabolomic profiling in improving preoperative risk stratification. However, implementing metabolomic profiling in routine clinical practice faces several challenges, including the high costs of LC-MS analysis, lack of standardization across analytical platforms, and limited accessibility in many healthcare settings. Also, future studies with larger cohorts of patients and integrated multi-omic approaches are needed to validate these biomarkers further and to explore the correlations between metabolic alterations and tumor aggressiveness.

## Figures and Tables

**Table 1:** Epidemiological and clinical characteristics of low-risk, high-risk, LNP, and LNN groups. Values represent medians and interquartile ranges. BMI—body mass index; FIGO—International Federation of Gynecology and Obstetrics staging system; LNP—lymph node-positive; LNN—lymph node-negative.

Group	Low-Risk	High-Risk	LNP	LNN
Number of patients	68	55	16	107
Age (years)	62 (58–66)	64 (59–68)	66 (63–70)	60 (57–64)
BMI (kg/m^2^)	32 (30–35)	28 (26–30)	28 (26–30)	31 (28–33)
FIGO stage				
IA	20	5	-	48
IB	8	6	-	16
II	4	9	2	8
III	6	38	14	32
IV		2	-	3
Grade				
1	24	17	3	48
2	13	16	5	31
3	1	27	8	28
Histology				
Endometrioid	68	45	10	94
Non-endometrioid	-	10	6	13

**Table 2:** The list of analytes differentiating between high-risk and low-risk endometrial cancer recurrence patients with *p*-values for each analyte. Abbreviations: C10—decanoylcarnitine; C12—Lauroyl-L-carnitine; LPC—lysophosphatidylcholine; PC—phosphatidylcholine; µM—micromolar.

Analyte [µM]	Whole Group *n* = 123	High-Risk Group *n* = 55	Low-Risk Group *n* = 68	*p*-Value Group 1 vs. 2
C10	0.16 (0.00–0.573)	0.12 (0.00–0.479)	0.19 (0–0.57)	0.0027
C12	0.06 (0.00–0.229)	0.06 (0.00–0.165.0)	0.07 (0–0.23)	0.05
Alanine	387.74 (198.0–651.0)	357.28 (224.0–613.0)	412.69 (198.0–651.0)	0.0087
Histidine	83.30 (45.7–146.0)	79.05 (45.7–118.0)	86.66 (49.5–146.0)	0.046
Tryptophan	52.05 (20.02–94.4)	48.04 (20.2–94.4)	55.32 (21.3–94.0)	0.049
Taurine	132.95 (71.6–226.0)	124.31 (71.6–175)	139.62 (75–226.0)	0.0064
LPC 16:1	1.97 (0.659–5.85)	1.78 (0.659–3.53)	2.11 (0.712–5.85)	0.03
PC 32:2	2.03 (0.584–4.51)	1.75 (0.649–3.82)	2.24 (0.584–4.51)	0.0045
PC 32:3	0.35 (0.132–0.665)	0.32 (0.132–0.665)	0.36 (0.178–0.62)	0.021
PC 34:3	9.95 (3.53–20.7)	9.00 (4.1–16.1)	10.69 (3.53–20.7)	0.014
PC 34:4	0.93 (0.322–2.17)	0.80 (0.322–1.69)	1.02 (0.41–2.17)	0.0093
PC 36:6	0.68 (0.223–1.99)	0.58 (0.223–1.38)	0.75 (0.261–1.99)	0.024
PC 38:0	2.52 (0.731–5.34)	2.24 (0.731–3.79)	2.75 (0.989–5.34)	0.02
PC 38:1	0.68 (0.00–2.17)	0.56 (0.00–1.24)	0.77 (0.00–2.17)	0.008
PC 38:3	37.92 (14.7–63.7)	34.83 (19.6–57.2)	40.37 (14.7–63.7)	0.016
PC 40:2	0.17 (0.064–0.418)	0.15 (0.064–0.26)	0.18 (0.07–0.42)	0.028
PC 40:3	0.36 (0.122–0.828)	0.33 (0.173–0.582)	0.38 (0.122–0.83)	0.021
PC O-36:3	4.51 (1.71–9.28)	4.17 (2.02–7.05)	4.79 (1.71–9.28)	0.03
PC O-36:4	13.51 (3.93–24.5)	12.51 (3.93–21.3)	14.34 (6.43–24.5)	0.039
PC O-38:0	1.95 (0.756–4.21)	1.75 (0.756–3.3)	2.11 (0.819–4.21)	0.046
PC O-38:5	12.54 (3.55–20.8)	11.60 (3.55–17.1)	13.31 (6.24–20.8)	0.034
PC O-38:6	5.83 (1.6–11.1)	5.25 (1.6–8.2)	6.28 (2.18–11.1)	0.031
PC O-40:1	0.88 (0.341–1.8)	0.78 (0.341–1.27)	0.96 (0.43–1.8)	0.018

**Table 3:** Concentrations of differentiating compounds between lymph node-negative (Group 1) and lymph node-positive (Group 2) patients with *p*-values for each analyte. Abbreviations: PC—phosphatidylcholine, µM—micromolar.

Analyte [µM]	Whole Group *n* = 123	Group 1 LNN Group *n* = 107	Group 2 LNP Group *n* = 16	*p* Group 1 vs. 2
Alanine	396.47 (198.0–651.0)	409.46 (198.0–651.0)	309.63 (245.0–433.0)	0.00000076
Asparagine	45.73 (22.3–68.0)	46.52 (22.3–68.0)	40.46 (32.3–57.7)	0.004
Histidine	84.65 (45.7–146.0)	85.97 (45.7–146.0)	75.85 (51.3–111.0)	0.034
Phenylalanine	76.39 (44.7–134.0)	77.15 (44.7–134.0)	71.34 (53.5–99.7)	0.016
Proline	173.85 (91.0–346.0)	177.09 (91.0–346.0)	152.16 (97.1–285.0)	0.0031
Threonine	118.00 (53.0–221.0)	120.12 (60.5–221.0)	103.81 (53.0–156.0)	0.035
Tyrosine	69.94 (31.2–126.0)	71.10 (31.2–126.0)	62.23 (49.3–104.0)	0.00018
PC 32:2	2.09 (0.6–4.6)	2.16 (0.6–4.6)	1.65 (0.8–2.7)	0.0069
PC 34:3	10.16 (3.5–20.7)	10.44 (3.5–20.7)	8.32 (4.1–15.3)	0.032
PC 34:4	0.96 (0.3–2.2)	0.99 (0.3–2.2)	0.78 (0.4–1.4)	0.016
PC 36:5	22.18 (5.2–50.2)	22.82 (8.2–50.2)	17.96 (5.2–36.8)	0.033
PC 36:6	0.71 (0.2–2.0)	0.73 (0.3–2.0)	0.57 (0.2–1.2)	0.017
PC 38:5	46.12 (18.4–74.7)	47.22 (18.4–74.7)	38.81 (20.5–63.5)	0.016
PC 40:5	8.24 (3.5–16.4)	8.50 (3.5–16.4)	6.54 (3.57–10.6)	0.0013
PC O-38:0	2.02 (0.8–4.2)	2.07 (0.8–4.2)	1.66 (0.873–3.27)	0.029

## Data Availability

The original contributions presented in this study are included in the article/supplementary material. Further inquiries can be directed to the corresponding author.

## Supplementary Materials

The following supporting information can be downloaded at https://www.mdpi.com/article/10.3390/metabo15070422/s1, Table S1. List of analytes with corresponding limit of detection (LOD) and coefficient of variation (CV) in QC2 samples.

## Abbreviations

The following abbreviations are used in this manuscript:

Ala	Alanine
Asn	Asparagine
ATX	Autotaxin
AUC	Area Under the Curve
C10	Decanoylcarnitine
C12	Lauroyl-L-carnitine
EC	Endometrial Cancer
ESGO	European Society of Gynaecological Oncology
ESMO	European Society for Medical Oncology
ESTRO	European Society for Radiotherapy and Oncology
FIGO	International Federation of Gynecology and Obstetrics
FIA-MS	Flow-Injection Analysis–Mass Spectrometry
HPLC-MS	High-Performance Liquid Chromatography–Mass Spectrometry
His	Histidine
IDO	Indoleamine-2,3-Dioxygenase
ISTD	Internal Standard
LC-MS	Liquid Chromatography–Mass Spectrometry
LNM	Lymph Node Metastasis
LPC	Lysophosphatidylcholine
MRM	Multiple Reaction Monitoring
PC	Phosphatidylcholine
Phe	Phenylalanine
PLS-DA	Partial Least Squares Discriminant Analysis
PORTEC-3	Post Operative Radiation Therapy in Endometrial Cancer 3 (study)
Pro	Proline
QC	Quality Control
ROC	Receiver Operating Characteristic
Thr	Threonine
Trp	Tryptophan
Tyr	Tyrosine
