# Supplementary material for: Targeted Metabolomic Serum Analysis of Patients with High and Low Risk of Endometrial Cancer Recurrence and Positive and Negative Lymph Node Status
Source: Metabolites. 2025 Jun 20;15(7):422. doi: 10.3390/metabo15070422 (PMC12298217; doi:10.3390/metabo15070422)
Supplement: Supplementary file 1 [file metabolites-15-00422-s001.zip › metabolites-3675434-supplementary.pdf]

Supplementary Materials

Table S1. List of analytes with corresponding limit of detection (LOD) and coefficient of variation (CV) in QC2 samples. C – acylcarnitine; LPC – lysophosphatidylcholine; PC – phosphatidylcholine; SM – sphingomyelin

Analyte	LOD (μM)	CV (%)
C0	4.00	5.4
C2	0.15	4.0
C3	0.08	4.6
C4	0.03	3.7
C5	0.04	6.8
C12	0.06	4.3
C12:1	0.20	4.2
C14:1	0.02	8.6
C16	0.02	5.1
C18	0.02	5.1
C18:1	0.04	6.8
C18:2	0.01	6.8
LPC 16:0	0.12	3.5
LPC 16:1	0.07	5.9
LPC 17:0	0.05	6.9
LPC 18:0	0.05	4.4
LPC 18:1	0.10	5.9
LPC 18:2	0.10	4.7
LPC 20:3	0.20	9.7
LPC 20:4	0.02	6.5
PC 28:1	0.04	7.9
PC 30:0	0.20	8.1
PC 30:2	0.01	30.2
PC 32:0	0.04	4.3
PC 32:1	0.06	3.9
PC 32:2	0.03	6.1
PC 32:3	0.01	8.1
PC 34:1	0.06	5.3
PC 34:2	0.10	5.0
PC 34:3	0.01	4.9
PC 34:4	0.01	5.4
PC 36:0	0.20	16.0
PC 36:1	0.03	5.2
PC 36:2	0.15	3.4
PC 36:3	0.04	3.9
PC 36:4	0.04	4.4
PC 36:5	0.01	5.2
PC 36:6	0.02	4.7

PC 38:0	0.20	5.8
PC 38:1	0.08	35.5
PC 38:3	0.04	2.9
PC 38:4	0.03	4.2
PC 38:5	0.02	3.6
PC 38:6	0.02	4.4
PC 40:2	0.02	6.4
PC 40:3	0.01	6.5
PC 40:4	0.01	4.0
PC 40:5	0.04	4.9
PC 40:6	1.20	4.2
PC 42:0	0.05	7.6
PC 42:1	0.01	10.2
PC 42:4	0.01	5.6
PC 42:5	0.05	11.9
PC 42:6	0.30	9.9
PC O-28:1	0.15	10.2
PC O-30:0	0.15	13.9
PC O-30:1	0.02	20.0
PC O-32:1	0.01	6.6
PC O-32:2	0.02	7.3
PC O-34:0	0.02	3.9
PC O-34:1	0.01	4.7
PC O-34:2	0.01	2.4
PC O-34:3	0.02	4.4
PC O-36:0	0.12	11.6
PC O-36:1	0.03	3.1
PC O-36:2	0.01	6.1
PC O-36:3	0.01	5.4
PC O-36:4	0.01	3.5
PC O-36:5	0.01	2.7
PC O-38:0	0.07	6.8
PC O-38:1	0.02	9.7
PC O-38:2	0.02	6.7
PC O-38:3	0.01	4.5
PC O-38:4	0.02	3.5
PC O-38:5	0.01	3.2
PC O-38:6	0.03	2.4
PC O-40:1	0.06	11.5
PC O-40:2	0.01	4.5
PC O-40:3	0.02	5.8
PC O-40:4	0.10	5.5
PC O-40:5	0.01	3.8
PC O-40:6	0.02	4.5
PC O-42:2	0.01	7.8
PC O-42:3	0.01	7.7
PC O-42:4	0.30	6.6
PC O-42:5	1.30	3.7

PC O-44:3	0.01	7.7
PC O-44:4	0.01	10.7
PC O-44:5	0.02	4.9
PC O-44:6	0.09	6.3
SM 33:1	0.02	6.7
SM 34:1	0.03	4.7
SM 34:2	0.01	4.0
SM 35:1	0.01	4.8
SM 36:1	0.07	4.3
SM 36:2	0.01	4.1
SM 38:3	0.00	14.6
SM 40:4	0.01	19.6
SM 41:1	0.02	4.0
SM 41:2	0.01	3.4
SM 42:1	0.13	3.3
SM 42:2	0.04	5.9
SM 43:1	0.01	9.2
SM 44:1	0.02	23.0
SM 44:2	0.01	12.6
Hexose	18.80	4.58
